# From Adhesions to Conception: A Case Study on Platelet-Rich Plasma’s Role in Gynecologic Recovery

**DOI:** 10.7759/cureus.86968

**Published:** 2025-06-29

**Authors:** Juncheng Zhang

**Affiliations:** 1 Department of Gynecology, Gansu Maternal and Child Health Care Hospital (Gansu Provincial Central Hospital), Lanzhou, CHN; 2 Department of Gynecology, Gansu University of Traditional Chinese Medicine, Lanzhou, CHN

**Keywords:** cold knife separation technique, fetation, hysteroscopy, intrauterine adhesions, platelet-rich plasma

## Abstract

Intrauterine adhesions (IUA) are a common gynecologic disorder usually caused by infection, surgery, or trauma. Its pathogenesis is complex and involves multiple factors such as cytokines, inflammatory responses, and tissue fibrosis. In recent years, diagnostic and therapeutic options for uterine adhesions have evolved, encompassing both traditional surgical methods and emerging biologic therapies. Platelet-rich plasma (PRP) therapy has gradually gained widespread attention in the field of obstetrics and gynecology. Its core concept is to utilize the abundant platelets in the patient’s own blood to promote tissue repair and regeneration. The application of PRP by intrauterine instillation or injection can effectively improve endometrial thickness, enhance its receptivity, and subsequently improve pregnancy outcomes.

In this paper, we report the case of a 33-year-old female patient who underwent hysterectomy for uterine remnants following a medical abortion. She subsequently developed secondary IUA, presenting as decreased menstrual flow. She initially received hysteroscopic cold knife adhesion separation combined with hormone replacement therapy, which proved ineffective. She later underwent repeat hysteroscopic cold knife adhesiolysis followed by intrauterine perfusion of PRP. After this intervention, her menstrual flow returned to normal, and she successfully conceived and delivered a healthy baby one year later.

## Introduction

Intrauterine adhesions (IUA), also known as Asherman's syndrome, are a common gynecological disease caused by endometrial injury or infection. It is pathologically characterized by adhesions in the uterine cavity, uterine isthmus, and cervical canal, and it severely affects women's fertility and quality of life. Although the exact prevalence of IUA is unknown, partly due to its insidious symptoms, lack of clinical awareness, and complex causative factors, studies have shown that its incidence is increasing [1]. The core etiology of the disease is closely related to intrauterine trauma and infection. Mechanical damage to the endometrium most commonly results from uterine surgery (e.g., curettage, abortion) or postoperative complications, while infection is often associated with inadequate postoperative care [2-3]. In clinical practice, the prevalence of IUA needs to be assessed in the context of various clinical presentations and surgical histories, including but not limited to secondary amenorrhea (prevalence 1-2%), infertility, recurrent miscarriage, postpartum hemorrhage (PPH), induced abortion, retained products of conception (RPOC), and surgical procedures involving manipulation of the uterine cavity (e.g., cesarean section, fibroid/adenoma myomectomy, diagnostic curettage, polypectomy, uterine teratoplasty, etc.) [1]. Notably, the prevalence of IUA varies significantly between clinical settings: it is lowest in patients with secondary amenorrhea (approximately 1-2%) and can be as high as 40-50% in women undergoing hysteroscopic myomectomy. Uterine adhesions are a common gynecological disorder that seriously affects women's reproductive health. Cold knife dissection combined with platelet-rich plasma (PRP) therapy has received widespread attention as an emerging therapeutic approach. The purpose of this article is to provide a comprehensive review of the basic theory, clinical practice, technological progress, epidemiology, controversial points, and future prospects of cold knife dissection combined with PRP for the treatment of uterine adhesions. Through a comprehensive analysis of relevant literature, the current research status of this treatment method in terms of its principles, efficacy, and safety is elaborated, and its challenges and future development directions are discussed to provide a reference for clinical application and further research.

## Case presentation

**Figure 1:**
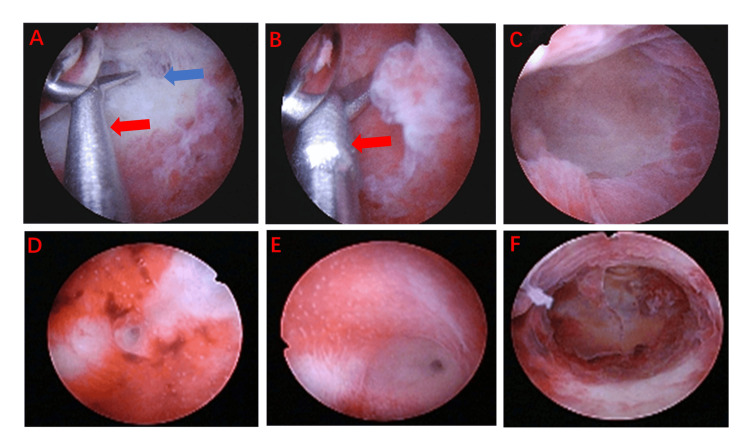
First inpatient procedure. Hysteroscopic cold-knife sharp adhesion disintegration performed in July 2022. Panels A, B, and C show the process of adhesion disintegration, while D, E, and F show the uterine cavity after disintegration. The blue arrow in A indicates the adhesion; the red marking indicates the disintegrating adhesion. The red arrow in B highlights the cold knife decomposing the adhesion.

**Figure 2:**
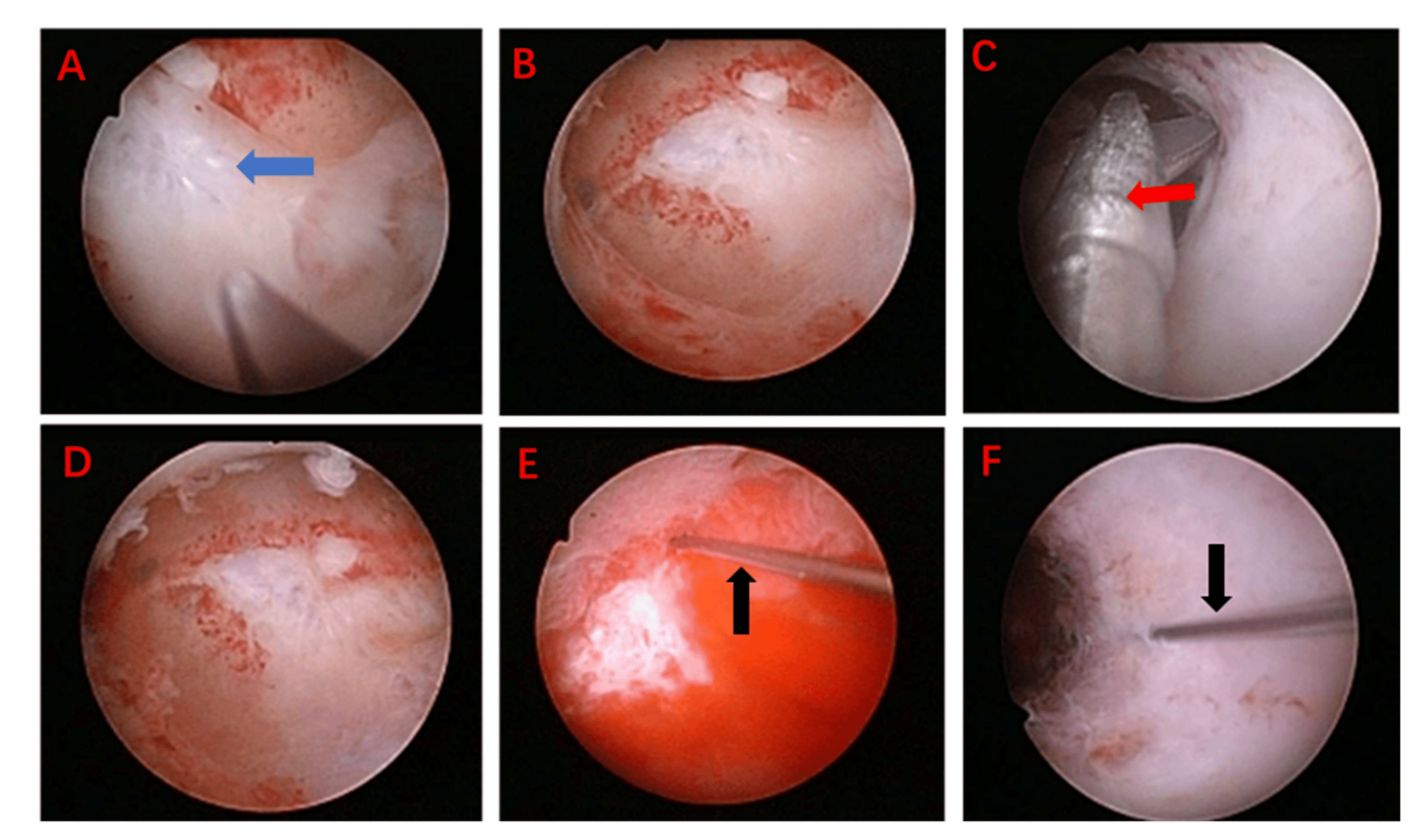
Surgical procedure of the second inpatient visit. Images show hysteroscopy with dissection of uterine adhesions, PRP subendometrial injection, and PRP intrauterine instillation as described in the text. Adhesions are marked in blue in panel A, decomposing adhesions are highlighted in red in panel C, and intrauterine PRP injection is indicated by black arrows in panels E and F. PRP: Platelet-rich plasma.

**Figure 3:**
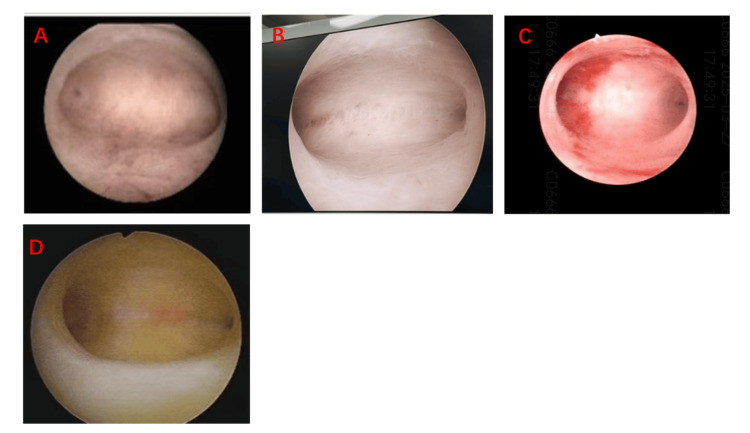
Examination chart of postoperative follow-up. Panels A, B, C, and D represent the patient's re-examination results at 1, 3, 6, and 24 months after the operation, respectively.

**Figure 4:**
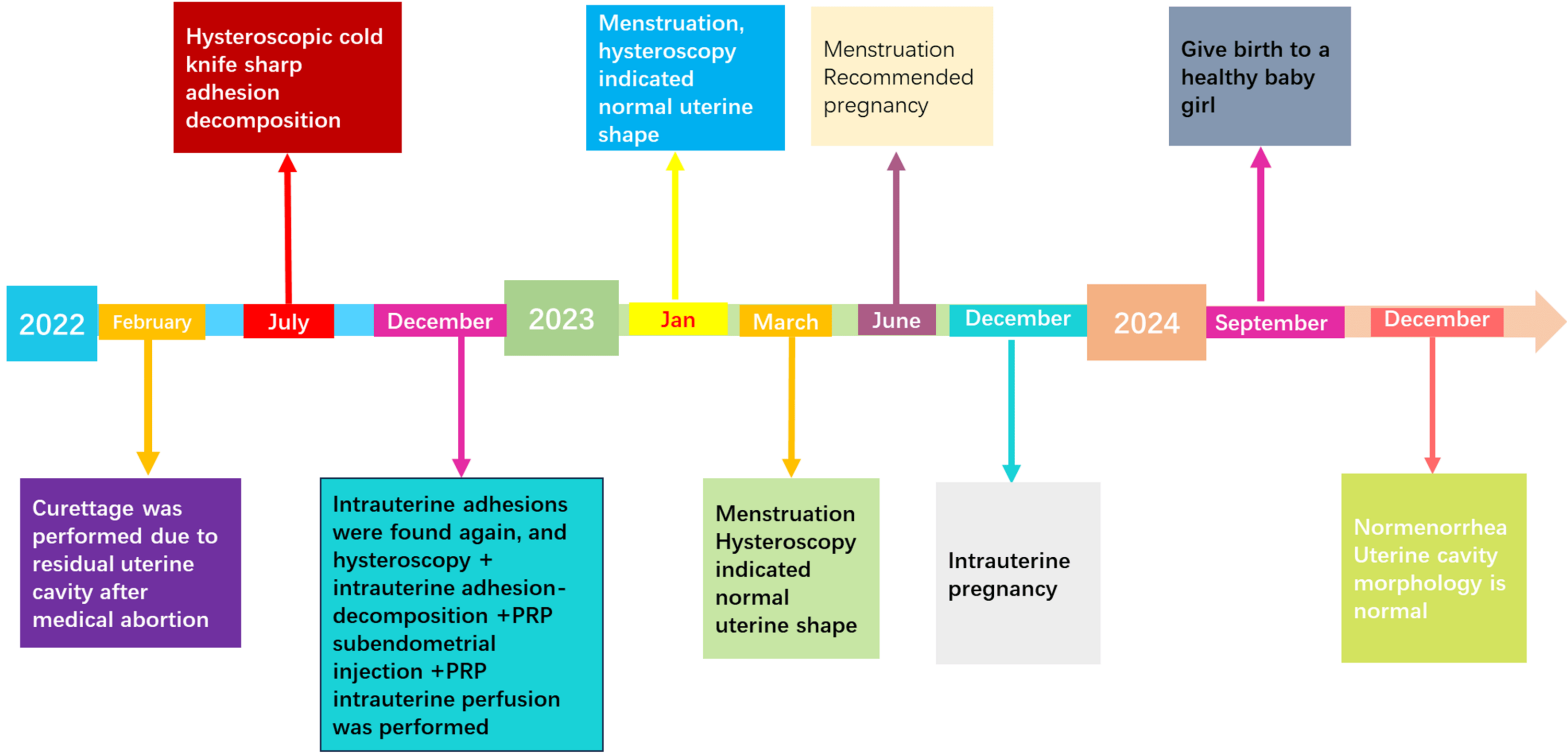
Flow chart of the patient's consultations and follow-up. The flow chart illustrates the patient's sequence of visits and treatments, clearly outlining the entire treatment process.

A 33-year-old female patient had a history of regular menstruation. In February 2022, she underwent a medication abortion. Due to residual tissue in the uterine cavity, she subsequently underwent uterine curettage. Following the procedure, she did not menstruate in March. She was able to menstruate after taking oral estrogen combined with progesterone; however, her menstrual flow was minimal, soaking only two sanitary napkins each time. In July 2022, she underwent hysteroscopic cold-knife sharp adhesion disintegration (the intraoperative situation is shown in Figure 1). Postoperatively, she was prescribed 2 mg of estradiol valerate orally, twice daily for 21 days. From the 15th day onward, she also took 100 mg of oral progesterone, two capsules once daily. In January, repeat hysteroscopy revealed a normally shaped uterine cavity. However, by the third month postoperatively, her menstrual flow gradually decreased, and menstruation was accompanied by irregular lower abdominal pain. In December 2022, a three-dimensional ultrasound suggested the following findings: (1) uneven endometrial thickness; (2) rounded bilateral uterine horns; and (3) possible adhesions. Given her desire for fertility, she was hospitalized in December 2022 for hysteroscopy, hysteroscopic adhesiolysis, PRP subendometrial injection, and PRP intrauterine instillation. During this hospitalization, the procedures were performed as shown in Figure 2. Intraoperative findings included: a smooth cervix; an approximately normal uterine cavity shape with thin endometrium; bilateral visualization of the fallopian tube ostia; keloidal fibrous tissue on the uterine floor; and fibrous adhesions on both lateral walls near the uterine horns. The adhesions were incised one by one using a cold knife to restore the normal uterine cavity shape. Under direct hysteroscopic and ultrasound guidance, 4 ml of PRP was injected at multiple points into the uterine wall, and an additional 6 ml was instilled into the uterine cavity. After the procedure, the patient was instructed to elevate her pelvis and remain in a static position for 10 minutes. Following surgery, the patient resumed regular menstruation. Postoperative evaluations in January, March, June, and two years later were all normal (as shown in Figure 3). She achieved a normal pregnancy within the first year post-surgery and has since delivered a healthy baby girl. All follow-up visits are summarized in Figure 4.

## Discussion

Pathophysiological mechanism of uterine adhesions

The pathophysiological mechanism of uterine adhesions is a cascade of abnormal repair following endometrial injury, centered on the vicious cycle of basal layer injury-fibrosis-regeneration imbalance. Based on recent research advances, this mechanism can be examined in depth across the following dimensions:

Basal Damage and Stem Cell Depletion

Mechanical damage to the basal layer of the endometrium, commonly due to intrauterine surgeries (e.g., abortion, curettage) or infections (e.g., *Mycobacterium tuberculosis*, HPV), leads to exposure of vascular endothelial cells and the release of profibrotic factors such as TGF-β1 and platelet-derived growth factor (PDGF). This activates platelets and recruits inflammatory cells. Repeated injuries significantly reduce basal stem cells (CD146+), resulting in impaired regenerative epithelialization and abnormal fibroblast colonization of the damaged area [3].

Imbalance of Fibrosis Regulatory Network

Pro-fibrosis factor storm: Injury localized TGF-β1 concentration is increased 10-20 fold, which activates fibroblast-to-myofibroblast differentiation via the SMAD2/3 pathway and upregulates the expression of α-smooth muscle actin (α-SMA) and type I/III collagen. Simultaneously, PDGF and connective tissue growth factor (CTGF) act synergistically to stimulate excessive extracellular matrix deposition [4]. Collapse of antifibrotic defenses: Interferon-gamma (IFN-γ) secretion is suppressed, and matrix metalloproteinase-9 (MMP-9) activity decreases by 50-70%, leading to a collagen degradation rate of less than one-third of normal [5]. Uncontrolled epithelial-mesenchymal transition (EMT): TGF-β1 triggers the downregulation of E-cadherin and upregulation of N-cadherin via the Wnt/β-catenin signaling pathway, thereby promoting the conversion of endometrial glandular epithelial cells into a mesenchymal phenotype [6].

Inflammation and Aberrations in the Immune Microenvironment

Abnormal macrophage polarization: the percentage of M1-type macrophages is increased to more than 75%, continuously releasing pro-inflammatory factors such as IL-6 and TNF-α, and activating the NF-κB pathway to exacerbate tissue damage [7]. Disruption of the Th17/Treg balance: an increased percentage of Th17 cells (>15%) drives the autoimmune response, while Treg cell function is suppressed by IL-2, and the immune tolerance mechanism fails [8]. Neutrophil extracellular traps (NETs): neutrophils in the infected state release NETs, whose histone H3Cit modification promotes fibrinogen cross-linking and the formation of physical adhesive scaffolds [9].

Impaired Vascular-Metabolic Reprogramming

Abnormal activation of hypoxia-inducible factor (HIF-1α): the partial pressure of oxygen in the injury zone decreases to <10 mmHg, and HIF-1α upregulates the VEGF inhibitor (sFlt-1), leading to a reduction in the density of neovascularization [10]. Abnormal mitochondrial metabolism: the fibroblast glycolysis rate is increased 3-fold (Warburg effect); lactate accumulation further acidifies the microenvironment and inhibits epithelial cell migration. Platelet-microthrombus storm: platelet aggregation in the injured area forms a microthrombus network that releases 5-HT and TXA2, leading to sustained vasospasm and ischemia-reperfusion injury [11].

Abnormal Epigenetic Regulation

Dysregulation of the miRNA regulatory network: miR-29 family (a collagen synthesis repressor) expression is decreased by 70%, while miR-21 (a pro-fibrotic miRNA) is increased 5-fold [12]. Abnormal histone modifications: H3K27me3 modification is enriched in the promoter regions of fibrosis-related genes (COL1A1, TGFBR2), maintaining pro-fibrotic episodic memory [13].

Innovative Perspectives

Recent studies suggest that "metabolic memory" in the endometrial basal layer may perpetuate fibrosis. Mitochondrial DNA released during injury activates the cGAS-STING pathway, inducing chronic type I interferon signaling and a profibrotic microenvironment. Single-cell sequencing has identified a CD9+ fibroblast subpopulation in adhesion sites that maintains a TGF-β autocrine loop, highlighting a potential therapeutic target. Collectively, these pathways outline the pathological axis: "Injury initiation-Inflammation amplification-Fibrosis stabilization-Regeneration inhibition," which provides a framework for targeted treatments (e.g., TGF-β inhibitors, epigenetic modulators) [14].

The pathophysiological mechanism of uterine adhesions is complex and involves a variety of factors. Trauma is a major cause, including procedures such as curettage, cesarean section, and other intrauterine surgeries, which can damage the basal layer of the endometrium and trigger a local inflammatory response. Inflammatory cells infiltrate the site and release various cytokines, such as transforming growth factor-β1 (TGF-β1). TGF-β1 activates the downstream Smad signaling pathway, promoting fibroblast proliferation and collagen synthesis. This leads to excessive fibrous tissue growth and the subsequent formation of adhesions.

Mechanism of action of PRP in tissue repair

PRP contains a variety of growth factors, including PDGF, TGF-β, vascular endothelial growth factor (VEGF), and many others [15]. These growth factors activate intracellular signaling pathways by binding to receptors on the cell surface, thereby promoting cell proliferation, migration, and differentiation. For example, PDGF stimulates the migration and proliferation of fibroblasts and smooth muscle cells and is involved in matrix synthesis and angiogenesis during tissue repair. VEGF primarily promotes the proliferation of vascular endothelial cells and neovascularization, which provides an adequate blood supply to the damaged tissue and accelerates the repair process [16]. Additionally, PRP helps regulate the inflammatory response. In the early stages of tissue injury, growth factors in PRP attract inflammatory cells to the injury site, initiate the inflammatory process, and facilitate the removal of damaged tissue and pathogens. As healing progresses, PRP inhibits excessive inflammation, preventing further tissue damage. At the same time, it promotes extracellular matrix synthesis and remodeling, creating a favorable microenvironment for cell adhesion, proliferation, and differentiation. In treating uterine adhesions, PRP supports the repair and regeneration of endometrial tissue through these mechanisms, reducing the formation of new adhesions and helping restore normal uterine function [15,16].

Growth Factor Release and Signaling Regulation

PRP’s core role arises from its concentration of over 30 bioactive factors, including PDGF, TGF-β, VEGF, IGF, and EGF [17]. These factors act through multiple mechanisms: first, by activating cell proliferation and differentiation, PDGF enhances fibroblast and endothelial cell proliferation to regenerate soft tissue, while TGF-β regulates osteoclast differentiation and collagen synthesis to support bone and cartilage repair. Furthermore, PRP exhibits a continuous release mechanism: 95% of pre-existing factors are released within 10 minutes of platelet activation, while new factors are synthesized and secreted by macrophages for up to 7 days, extending the repair cycle.

Fibrin Scaffold Construction

Fibrin in PRP also forms a localized three-dimensional mesh structure [18]. This structure provides physical support for cell migration and proliferation and prevents the loss of platelets and repair factors. It also guides tissue remodeling by promoting wound contraction and directional alignment of vascular endothelial cells to accelerate wound closure.

*Anti-inflammation and Immunomodulation* 

Inhibition of excessive inflammation: High concentrations of anti-inflammatory factors such as TGF-β and IL-10 inhibit pro-inflammatory cytokines (e.g., TNF-α) and reduce tissue damage. Antimicrobial and immune defense: Leukocytes (e.g., neutrophils, monocytes) in PRP directly phagocytose pathogens and disrupt bacterial membrane structure through antimicrobial peptides [19].

Vascular Neovascularization and Microcirculation Improvement

Growth factors such as VEGF and PDGF stimulate the proliferation and migration of vascular endothelial cells, promoting capillary angiogenesis. This enhances blood flow to the site of injury, improves oxygen and nutrient delivery, facilitates removal of metabolic waste, and ultimately creates an optimal environment for tissue regeneration and healing [20].

Clinical practice of cold knife separation combined with PRP in the treatment of uterine adhesions

The clinical efficacy of cold knife separation combined with PRP in the treatment of uterine adhesions has been analyzed in several studies. In one study [20], the clinical efficacy of hysteroscopic intrauterine PRP injection was found to be superior to intrauterine PRP infusion in patients with moderate-to-severe IUAs. This approach significantly reduced adhesion scores, improved menstruation, and increased clinical pregnancy rates. A total of 299 patients with moderate-to-severe IUA who were treated with PRP after transcervical resection of uterine adhesions (TCRA) were divided into two groups: those receiving subendometrial PRP injection and those receiving intrauterine PRP infusion. The primary outcome was the clinical pregnancy rate, while secondary outcomes included menstrual improvement and changes in AFS scores. Results showed that the reduction in AFS score was greater in the injection group than the infusion group (8 vs. 7, p = 0.019); the menstrual improvement rate was also higher in the injection group (77.0% vs. 52.9%, p < 0.001). Although the clinical pregnancy rate did not differ significantly between groups (28.4% vs. 20.4%, p = 0.208), multivariate logistic regression revealed a significantly higher pregnancy rate in the injection group (OR = 2.020, 95% CI = 1.050-3.889, p = 0.035). Propensity score matching (PSM) analysis further showed that the median reduction in postoperative AFS scores was significantly higher in the injection group than in the perfusion group (p = 0.015); the improvement in menstruation was also greater (75.0% vs. 58.1%, p = 0.027), and the clinical pregnancy rate was significantly higher (29.4% vs. 16.2%, p = 0.043). In another meta-analysis [21] that included 12 studies, eight randomized controlled trials (RCTs) and four non-RCTs, with a total of 874 participants (425 in the PRP group and 449 in the control group), PRP administration significantly reduced the recurrence of moderate-to-severe adhesions (RR: 0.477, p < 0.001), lowered AFS scores (MD: 0.862, p = 0.043), improved clinical pregnancy rates (RR: 1.373, p = 0.036), and increased menstrual flow (MD: 2.956, p < 0.001). However, there were no significant differences in biochemical pregnancy, miscarriage, or live birth rates.

Technical advancements in cold knife separation combined with PRP for the management of uterine adhesions

Optimization and Standardization of PRP Preparation Techniques

Currently, various methodologies are employed for the preparation of PRP, with each method influencing the platelet concentration, growth factor content, and platelet activity within the PRP. To achieve standardization, researchers have undertaken comprehensive studies focusing on parameters such as centrifugation speed, duration, and anticoagulant usage. Notably, it has been demonstrated that employing a double centrifugation process significantly enhances the quantity and yield of platelets in PRP while minimizing contamination by red blood cells and leukocytes. This renders double centrifugation particularly advantageous for both autologous and allogeneic PRP preparation [22]. Furthermore, research indicates that the rate and duration of centrifugation are critical parameters affecting platelet yield, with lower centrifugation speeds and shorter durations resulting in higher platelet counts and enrichment percentages [23,24].

Development of a Comprehensive Treatment Plan

The formulation of an effective and comprehensive treatment plan for the integration of cold knife dissection with PRP therapy is crucial for enhancing therapeutic outcomes in cases of uterine adhesions. Prior to surgical intervention, a thorough evaluation of the patient is essential, encompassing the assessment of the severity and classification of uterine adhesions, as well as the patient's reproductive aspirations. This evaluation is imperative for creating a tailored treatment strategy. In instances of mild uterine adhesions, direct PRP injection following cold knife dissection can facilitate endometrial repair and regeneration. Conversely, for patients presenting with moderate-to-severe uterine adhesions, it may be necessary to incorporate additional adjunctive therapies, such as the insertion of intrauterine devices or the administration of estrogen following cold knife dissection. These measures, in conjunction with PRP, aim to synergistically minimize the risk of re-adhesion [25]. During surgical procedures, it is imperative to meticulously regulate the extent and scope of cold knife separation to prevent undue harm to the endometrium. Concurrently, precise timing and dosage of PRP injection are essential. Empirical evidence suggests that administering an appropriate quantity of PRP either immediately or shortly after cold knife separation significantly enhances its efficacy in facilitating tissue repair. Furthermore, postoperative follow-up and monitoring are critical components of patient care. Regular ultrasonography and hysteroscopy enable continuous assessment of uterine cavity recovery, allowing for tailored adjustments to the treatment plan based on the patient's specific condition, thereby ensuring the effectiveness and safety of the comprehensive therapeutic approach.

Indications and contraindications for cold knife dissection combined with PRP therapy

The primary indications for the use of cold knife dissection in conjunction with PRP therapy are as follows [26]: (1) Cervical adhesions or cervical canal stenosis, particularly in cases of moderate-to-severe adhesions. In such instances, the loosening of adherent tissues through cold knife surgery followed by the local injection of PRP can expedite tissue repair and decrease the likelihood of postoperative re-adhesions. (2) Chronic cervicitis associated with impaired tissue repair, where the growth factors present in PRP facilitate mucosal regeneration. (3) Postoperative treatment for benign cervical lesions, such as after cervical polypectomy or loop electrosurgical excision procedure (LEEP), where PRP adjuvant therapy can mitigate scar formation and enhance cervical function.

The contraindications for this combined therapeutic approach include: (1) Active infections of the reproductive tract, such as acute cervicitis or active HPV infection, which may exacerbate inflammation or result in the failure of PRP therapy; (2) Patients with abnormal coagulation function or thrombocytopenia, who may experience limited efficacy from PRP treatment as its preparation relies on autologous platelets; (3) Individuals with cervical malignancies, particularly those previously treated with PRP adjuvant therapy, who may be ineligible for further PRP use. Additionally, in cases of cervical malignant tumors or pre-cancerous lesions (e.g., CIN II-III without standardized treatment), procedures such as cold knife surgery may facilitate disease dissemination, and the growth-promoting properties of PRP could potentially accelerate tumor progression. (4) Pregnancy or breastfeeding, as PRP treatment may interfere with pregnancy status. (5) Allergies to PRP components or the presence of immune system disorders, which may provoke abnormal immune responses. In clinical practice, it is imperative to thoroughly assess the pathological stage and systemic condition of patients, ensuring the exclusion of malignant lesions through cervical biopsy prior to initiating combination therapy.

Controversial aspects of cold knife separation combined with PRP in the management of uterine adhesions

Evaluation of Safety and Risk in Cold Knife Separation Combined With PRP

The safety and risk assessment of cold knife separation combined with PRP therapy is a significant concern within the medical community. From a surgical perspective, although cold knife dissection offers a relatively precise approach, it is not without risks, including potential uterine perforation and hemorrhage. However, advancements in surgical techniques and accumulated clinical experience have contributed to reducing the incidence of such complications. For instance, one study reported a low incidence of severe complications such as uterine perforation [27]. Regarding PRP therapy, despite being derived from autologous blood, which theoretically minimizes the risk of immune rejection, there are inherent potential risks. The PRP preparation process requires strict aseptic conditions to prevent infection. Furthermore, the possibility of over-activation or aberrant activity of growth factors within PRP may pose adverse effects on tissues. Nevertheless, the majority of contemporary studies suggest that the combination of cold knife dissection with PRP therapy generally maintains a favorable safety profile.

Debate on the Efficacy of PRP in Treating Uterine Adhesions

The efficacy of PRP in treating uterine adhesions remains a topic of debate. Several studies have shown that PRP may facilitate endometrial repair and regeneration, thereby improving reproductive outcomes in patients with uterine adhesions. For example, clinical investigations have demonstrated that patients undergoing cold knife dissection combined with PRP exhibit improved menstrual recovery and increased clinical pregnancy rates [28]. However, other studies have questioned these findings. It has been proposed that the therapeutic efficacy of PRP may be influenced by factors such as the method of preparation, dosage, and timing of administration. Variations in these techniques can result in inconsistent concentrations and activity levels of growth factors, potentially affecting outcomes. Moreover, small sample sizes in some studies may limit the reliability of their conclusions. Therefore, larger, high-quality studies are needed to more definitively evaluate the effectiveness of PRP in this context.

Long-term Outcomes and Recurrence Rates of Cold Knife Dissection Combined With PRP Treatment

The long-term outcomes and recurrence rates associated with cold knife dissection combined with PRP therapy are also important clinical considerations. Current evidence suggests that this combination can alleviate symptoms of uterine adhesions and improve fertility outcomes within a specific timeframe. Follow-up studies have shown that many post-surgical patients experience normalization of menstruation, improved uterine morphology, and, in a notable number of cases, successful conception and delivery. Nonetheless, questions remain regarding the durability of these improvements and the risk of recurrence. Extended follow-up periods have revealed cases of re-adhesion, potentially compromising long-term efficacy. Recurrence may be attributed to various factors, including individual differences in healing, postoperative care practices, and the regenerative capacity of the endometrial tissue [29]. Some studies also suggest that insufficient restoration of the local endometrial microenvironment following surgery could play a critical role in the development of re-adhesion [30].

Future prospects of cold knife separation combined with PRP in the treatment of uterine adhesions

Potential New Applications of Cold Knife Separation Combined With PRP

The integration of cold knife separation with PRP therapy holds promising potential for novel applications in the future. As our understanding of the pathophysiological mechanisms underlying uterine adhesions deepens, this combined therapeutic approach may be extended to address a broader spectrum of related conditions. For instance, in cases of menstrual disorders and infertility resulting from endometrial damage, the synergy of cold knife separation and PRP therapy could offer innovative treatment alternatives by facilitating endometrial repair and alleviating disease symptoms. Furthermore, this combined treatment approach may exhibit significant potential for advancement within the realm of regenerative medicine. By integrating tissue engineering techniques and harnessing the reparative properties of PRP, more efficacious materials for endometrial regeneration could be developed. For example, the amalgamation of PRP with bioscaffold materials could lead to the creation of biologically active endometrial substitutes, offering therapeutic solutions for conditions such as severe uterine adhesions or compromised endometrial integrity.

Emerging Research Directions and Challenges in Cold Knife Separation Combined With PRP

Emerging research directions in the integration of cold knife separation with PRP therapy include further optimization of treatment protocols and the investigation of novel mechanisms of action. In the realm of protocol optimization, researchers are focused on identifying the optimal operational parameters for cold knife dissection, as well as determining the most effective dosage and timing for PRP administration. For instance, multicenter, large-sample clinical trials have been conducted to ascertain the ideal dosage and time intervals for PRP injections in patients with varying degrees of uterine adhesions, with the aim of enhancing therapeutic efficacy [31]. Nevertheless, the field encounters several challenges. Firstly, the preparation and quality control of PRP require further standardization. Currently, discrepancies in PRP preparation methods across different studies and clinical applications hinder the uniform assessment of its therapeutic effects. Secondly, there is a need for more long-term follow-up studies to validate the sustained safety and efficacy of combining cold knife dissection with PRP therapy.

## Conclusions

The presentation of this case is novel and offers an interesting perspective for women with intrauterine adhesions associated with infertility. One limitation is that it involves only a single case; the next step would be to compile a case series and work toward establishing standardized management protocols.
